# Efficient encoding of motion is mediated by gap junctions in the fly visual system

**DOI:** 10.1371/journal.pcbi.1005846

**Published:** 2017-12-04

**Authors:** Siwei Wang, Alexander Borst, Noga Zaslavsky, Naftali Tishby, Idan Segev

**Affiliations:** 1 Department of Neurobiology, Hebrew University Jerusalem, Jerusalem, Israel; 2 Max Planck Institute of Neurobiology, Martinstried, Munich, Germany; 3 The Edmond and Lily Safra Center for Brain Sciences, Hebrew University Jerusalem, Jerusalem, Israel; 4 Department of Computer Science, The Hebrew University of Jerusalem, Jerusalem, Israel; Université Paris Descartes, Centre National de la Recherche Scientifique, FRANCE

## Abstract

Understanding the computational implications of specific synaptic connectivity patterns is a fundamental goal in neuroscience. In particular, the computational role of ubiquitous electrical synapses operating via gap junctions remains elusive. In the fly visual system, the cells in the vertical-system network, which play a key role in visual processing, primarily connect to each other via axonal gap junctions. This network therefore provides a unique opportunity to explore the functional role of gap junctions in sensory information processing. Our information theoretical analysis of a realistic VS network model shows that within 10 ms following the onset of the visual input, the presence of axonal gap junctions enables the VS system to efficiently encode the axis of rotation, θ, of the fly’s ego motion. This encoding efficiency, measured in bits, is near-optimal with respect to the physical limits of performance determined by the statistical structure of the visual input itself. The VS network is known to be connected to downstream pathways via a subset of triplets of the vertical system cells; we found that because of the axonal gap junctions, the efficiency of this subpopulation in encoding θ is superior to that of the whole vertical system network and is robust to a wide range of signal to noise ratios. We further demonstrate that this efficient encoding of motion by this subpopulation is necessary for the fly's visually guided behavior, such as banked turns in evasive maneuvers. Because gap junctions are formed among the axons of the vertical system cells, they only impact the system’s readout, while maintaining the dendritic input intact, suggesting that the computational principles implemented by neural circuitries may be much richer than previously appreciated based on point neuron models. Our study provides new insights as to how specific network connectivity leads to efficient encoding of sensory stimuli.

## Author summary

Understanding sensory stimuli from the environment and deciding how best to respond to it behaviorally is essential for survival. What makes organisms efficient in encoding these sensory stimuli? This study provides a novel view on this unresolved issue using the visual system of the fly. We show that a specific synaptic connectivity manifested via gap junctions (GJs) among axons in the Vertical System (VS) network leads to particularly high encoding efficiency of the axis of rotation of the fly’s ego motion. Due to these GJs, triplets of VS neurons (the VS5-6-7 triplet), which connect to a downstream motor system, encode motion stimuli at an efficiency close to the physical limit; this efficient encoding is necessary for evasive maneuvers that are critical for the fly to escape predators. We then suggest why GJs in the VS network enable such high encoding efficiency.

## Introduction

The principles governing synaptic connectivity may hold the key to understanding the functional organization of the brain. Gap junctions (GJs) underlying the operation of electrical synapses are found in both the central nervous system [1–4] and the sensory system [5,6,7]. However, their functional role still remains elusive despite extensive studies [8–13]. Here we address this problem in the vertical system (VS) network of the fly visual system, where recent studies [14–18] have identified that in this sensory network, individual neurons primarily use GJs to communicate with one another. This provides a unique opportunity to characterize the function of GJs in the context of sensory information processing.

In the visual system of the blowfly *Calliphora vicina*, photoreceptor signals are processed in four consecutive layers of the neuropile: the lamina, medulla, lobula, and lobula plate, each of which is arranged in columnar, retinotopic fashion. In a striking parallel to the vertebrate retina [19, 20], the direction of visual motion is computed in parallel ON and OFF motion pathways [21]. Columnar T4 and T5 cells in the lobula and lobula plate, represent the output of the ON and the OFF pathway, respectively [22]. They synapse onto the dendrites of large-field tangential cells (LPTC) such as the horizontal system (HS) and vertical system (VS) cells [23, 24, 25]. Among these LPTCs, 20 different VS cells have been described. There are 10 VS cells in both the left and right compound eyes, ordered VS1 to VS10 along the anterior-posterior axis [26]. These were shown to encode the azimuth degree of the axis of rotation via their axonal voltages (these are non-spiking neurons). VS cells are connected to other LPTC cells within the lobula plate, as well as to downstream neurons such as the descending neurons (DNOVS1, 2 [26, 27]) that are upstream to the neck motor center. Intriguingly, adjacent VS cells are connected to each other via axonal GJs, whose conductance is on the order of 1 μS [16,18]. Surprisingly, the only identified output of the VS system to the downstream system is the connection between the left and the right VS 5-6-7 triplets to the DNOVS neurons. No downstream pathways are known to read out from the whole VS 1–10 network.

The VS network is an early sensory network that encodes motion information from a complex environment under severe constraints. For example, the fly only requires a 30–40 ms visual-motor delay to elicit evasive maneuvers [28, 29] to escape from swats effectively. Previous work hypothesized that this is because neural coding of sensory information is efficient [30–37]. This principle has been used successfully to account for many observed properties in sensory systems, from the size and shape of receptive fields [30, 31, 38, 39], and the statistics of the spike train [40, 41], to the higher-order interaction of populations of neurons [42–46]. However, previous work on efficient coding with population of neurons has not considered whether the inclusion of a specific connectivity feature i.e., GJs, can contribute to the emergence of this encoding efficiency [47].

Here, we investigated whether the VS network encodes motion information efficiently using axonal GJs and if so, the implications of this efficient encoding of motion in behavioral contexts. However, because experimental studies that record the impact of the visual input impinging on VS cell dendrites usually use calcium imaging [48], the response of VS cell dendrites at millisecond precision is still unavailable. We therefore investigated this problem by using a physiologically realistic model of the VS network [16]. Because previous work had shown that GJs can only help encoding when they are located at the axons [17], here we contrast with and without axonal GJs in this VS network to quantify the effect of the axonal GJs. In addition, because this network connects to the neck motor center via descending neurons (DNOVS1,2) and DNOVS2 has a maximum firing rate of 100Hz ([27], DNOVS1 is a graded neuron), we used the first 10 ms after the stimulus onset to sample the VS axonal voltages.

Based on our simulations of the model VS network with GJs and without GJs in two different stimulus conditions (natural and checkerboard), below we show that these axonal GJs enable VS cells to reduce the fluctuation in encoding individual axes of rotation, such that VS cells can jointly encode different axes of rotation with better separability. Furthermore, our information theoretic analysis showed that with GJs, motion encoding by the VS network, based on its joint axonal output voltage, extracts almost all the available motion information provided by dendritic input. We also found that the VS 5-6-7 triplet which is directly connected to downstream DNOVS1,2 neurons encodes motion more efficiently than the network as a whole, and reaches at least 90% of the physical limit of performance as determined by the statistical structure of the visual input itself. This near-optimal encoding efficiency emerged as robust regardless of the signal to noise ratios. Because GJs are formed among the axons of VS cells, they only impact the system’s readout while maintaining the dendritic input intact, suggesting that the computational principles implemented by neural circuitries may be much richer than previously assumed from point neuron models [49, 50]. Finally, we demonstrate that this efficient encoding of motion enabled by the GJs is behaviorally important. Without GJs, the VS network cannot even encode some basic rotations correctly such as the up/down tilt of the head and body (pitch rotation). Considering that evasive maneuvers involve a banked turn [29, 51] which combines roll and pitch at the same time, this finding suggests that the presence of GJs is critical for the survival of the fly.

## Results

### Assessing the role of gap junctions (GJs) in encoding motion stimuli in the fly VS network

**Fig 1:**
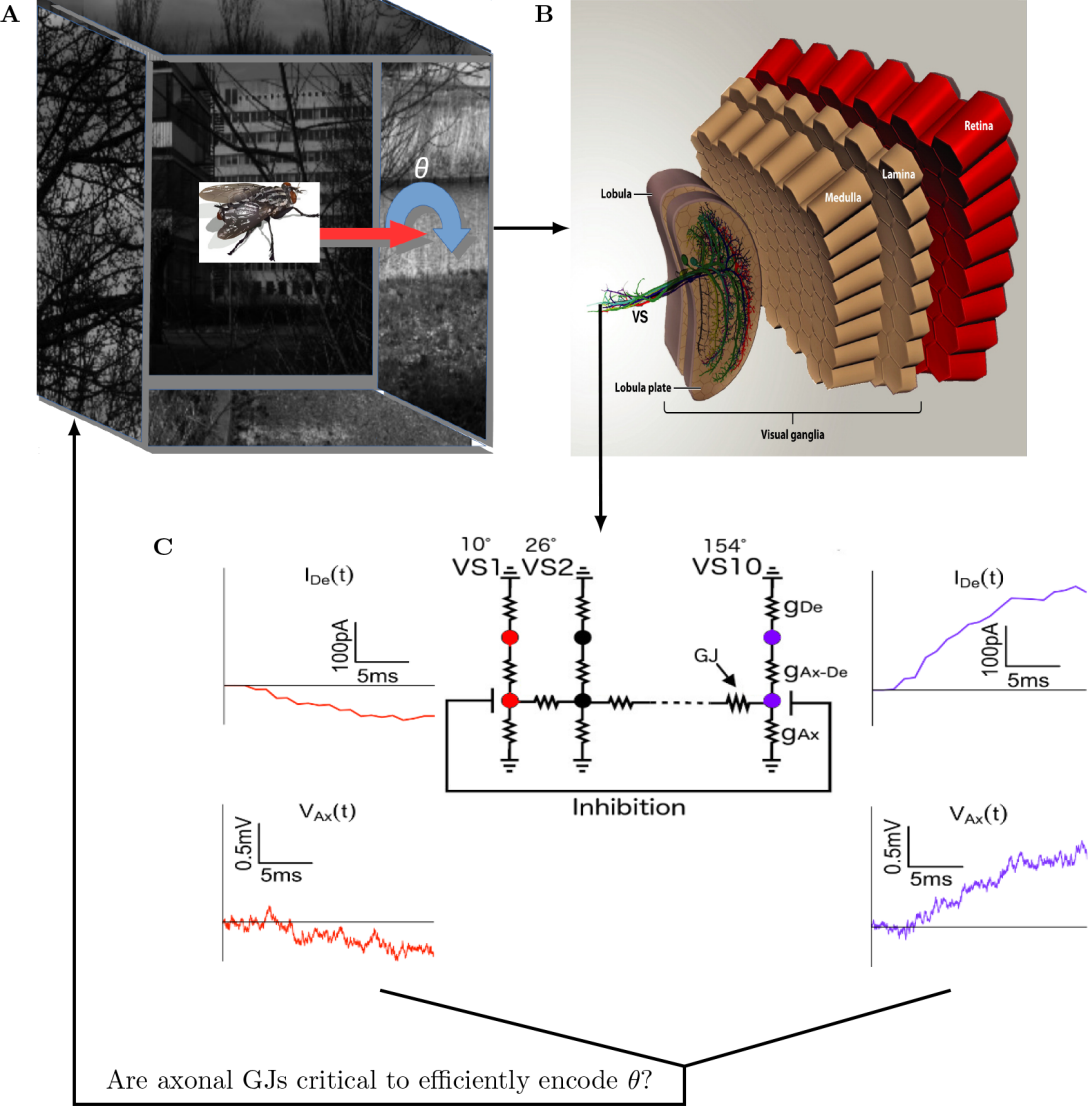
How do axonal GJs help to efficiently encode the axis of ego rotation, *θ*, in the VS network of the fly? (**A**) Schematic depiction of the visual stimuli used in the simulation. Six natural images (five are shown here except the frontal one) were randomly selected from the Van Hateren and Schilstra dataset [51]; each image was patched on a different face of a cube. Assuming that the fly is located in the center of this cube, we obtain the optic flow pattern of the fly’s ego rotation around *θ* (thick blue arrow) by rotating this cube around *θ*. (**B**) The fly visual system is composed of a retina, lamina, medulla, lobula and lobula plate. The retina, lamina and medulla are organized retinotopically. The vertical system (VS) network in the lobula plate integrates output from the upstream LMD units and sends global motion-sensitive signals downstream. (**C**) The VS model used in the present study; in this figure, the 10 VS cells of the right visual system are shown. In this model, the complex dendritic branches of the VS cells are reduced to a single compartment (g_De_); this dendritic compartment is connected via an axial resistance to the axonal compartment (g_Ax-De_)_._ The VS cells are connected to each other sequentially via axonal GJs; each VS has a preferred dendritic receptive field (RF) center (e.g., 10° for VS1, 26° for VS2 and 154° for VS10, as indicated). The computed dendritic input following visual input are shown in red and purple for VS1 and VS10, respectively. The corresponding axonal voltages (V_Ax_) are also shown. In this work, we only used the first 10 ms of the dendritic input and the axonal output.

To investigate how/whether axonal GJs in the VS network impact the encoding efficiency of the axis of rotation θ, we followed the procedure depicted in Fig 1. Fig 1A shows an example stimulus, which has a fixed axis of rotation embedded in a cube (the “cage”) with a randomly generated natural scene. We mimicked the optic flow of a fly rotating in the center of this “cage” by rotating the cage accordingly (see **Materials and Methods**). This optic flow was projected to the fly visual system (Fig 1B), thus generating responses from approximately 5000 local motion detectors (LMD, not shown, see **Materials and Methods**) following the retinotopic organization from the retina through the lobula [52]*)*. The output of these LMDs were then projected to the dendrites of VS cells (detailed morphology is depicted in the lower left of Fig 1B).

Fig 1C shows the physiologically realistic and simplified VS network model used in this study. In this model, each VS neuron is represented by a single dendritic compartment connected via an axial resistance to an axonal compartment. The dendritic compartment integrates the current generated by all LMDs which are located within the receptive field (RF) of that cell. The RF was defined as a 2D Gaussian with an azimuth width of 15° and an elevation width of 60° (see **Materials and Methods**). The dendritic compartments of the VS neurons differed as regards the center of their RFs; i.e., the center of the RF of VS1 was at 10°, but was at 26° for VS2 and 154° for VS10 (Fig 1C top values, and see also Table 1 in **Materials and Methods** for a summary of the RFs of all 10 VS cells of the right compound eye, the RFs of the VS cells in the left compound eye are symmetrically located at -10° for VS1, …, -154° for VS10, respectively). The traces in Fig 1C show an example of the input current to the dendrites (top trace) and the corresponding axonal voltage (lower trace) following a visual input, for VS1 (in red) and VS10 (in purple), respectively. As can be seen, the axonal voltages of these VS neurons do not exactly reflect the corresponding input current to their dendritic compartment, i.e. note that while the current is relatively smooth, the axonal voltage of these VS neurons shows much larger jitter; at one point, it even flips sign to be opposite to the sign of the dendrite current (compare the two red traces at left of Fig 1C).

**Table 1:** The centers of dendritic RF for VS1-10 (right eye).

VS1	VS2	VS3	VS4	VS5	VS6	VS7	VS8	VS9	VS10
10°	26°	42°	58°	74°	90°	106°	122°	138°	154°

This implies that the effect of the axonal GJs (the current flowing via these GJs between adjacent axons) was significant. The arrow connecting Fig 1C to Fig 1A depicts the key aim of this study, which was to identify, using the information bottleneck method [53] as our major theoretical approach, how connectivity features such as these axonal GJs in the VS system give rise to efficient encoding of the visual input in a realistic visual scenario. We only used the axonal voltage for the first 10ms it seems that this is the relevant timescale for integration in the VS network (see S2 Fig, which shows that a longer integration window does not help to encode additional motion information).

### GJs improve the separability of the axes of rotation

**Fig 2:**
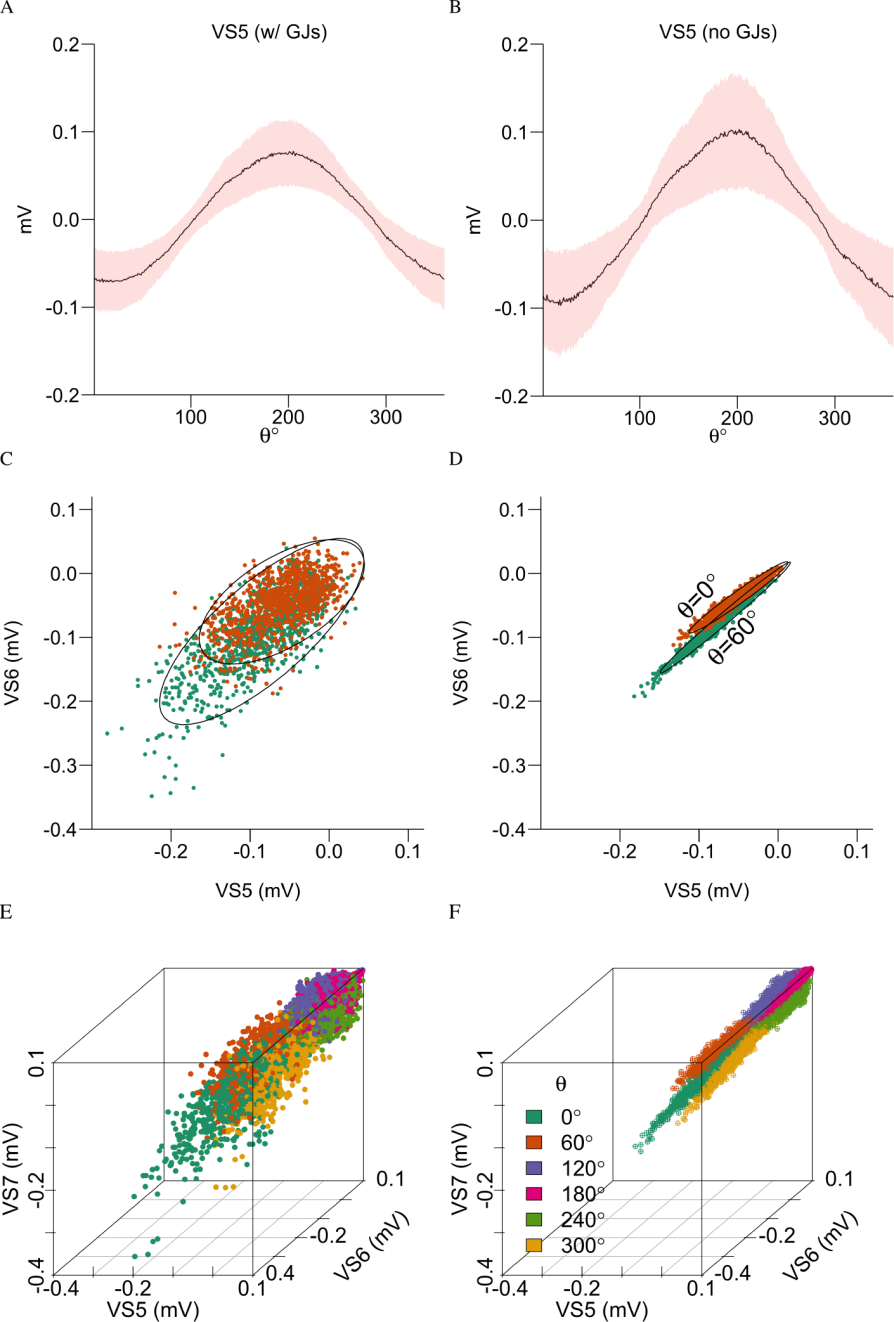
GJs reduce the variability and enhance the clustering of the combined axonal responses to different axes of rotation. (**A**) Response of VS5 to stimuli embedded with natural scenes as a function of the rotation axis when GJs are absent from the VS network. The continuous line shows the mean voltage response; the pink shaded area represents one standard deviation from that mean. (**B**) As in **A** but when the VS cells are all connected with GJs = 1 μS (see the circuit in Fig 1C). (**C**) Joint axonal voltage response of VS5 versus VS6 in the absence of GJs. A total of 1000 samples for both *θ* = 0° (green) and for *θ* = 60° (red) in response to natural stimuli are shown (see **Materials and Methods**). Their 95% confidence ellipses are shown in black. (**D**) As in **C** but with GJs = 1 μS. (**E**) Joint axonal voltages for VS5-6-7 of the left compound eye without GJs and (**F**) with GJs = 1 μS for six different axes of rotation (indicated by respective colors). Note the greatly improved separability of the axes of rotation in the presence of GJs.

In Fig 2 we showed that GJs among axons in the VS system help the joint axonal voltage to obtain better clustering with respect to different axes of rotation, *θ*. We first computed the voltage response of various VS cells separately to natural scenes as a function of the axis of rotation, both with and without axonal GJs (Fig 2A and 2B, respectively for an example VS5 cell). We found that with axonal GJs, both the range and variability of the VS cell voltage responses were smaller than without GJs. We next investigated, in Fig 2C and 2D, how the reduced variability due to the axonal GJs, as found for single neurons (Fig 2A and 2B) influences the joint axonal response of two VS cells. To this end, we plotted the voltage response (as well as the 95% confidence ellipses of the joint voltages) induced by the natural scene of VS5 versus VS6 for two axes of rotation (*θ* = 0° in green and *θ* = 60° in red). Thus, the inclusion of GJs strengthened the correlation between the joint axonal voltages (compare Fig 2C with Fig 2D) and, consequently, reduced the overlap in the joint axonal responses to the different axes of rotation. Without GJs, the 95% confidence ellipses for 0° and 60° almost entirely lay on top of each other whereas with GJs the corresponding 95% confidence ellipses only have a small area of overlap. This result suggests that the joint voltage responses of VS5 and VS6 form distinct clusters for different axes of rotation with axonal GJs but not without GJs.

When the joint voltage responses of the triplets (e.g., VS5-6-7) of cells were computed, we found that with axonal GJs, the encoding of the full range of the axes of rotation was dramatically improved (comparing Fig 2E to Fig 2F). The presence of GJs reduced the variability even further in with higher dimensions, while enhanced the linear correlation among the voltage responses of the VS5-6-7 cells, resulting in joint voltages that were tightly clustered for each axis of rotation, with little overlap between axes of rotation for the different cells (Fig 2F). This distinct clustering was hardly visible when GJs were absent (Fig 2E). As we further showed in S4 Fig in more detail, both the reduced variability the strengthened correlation induced by the axonal GJs led to superior encoding of the axis of rotation, *θ*, which was characterized by improved precision in mapping *θ* to distinguishable clusters of the joint VS responses.

### GJs enable near-optimal motion encoding

**Fig 3:**
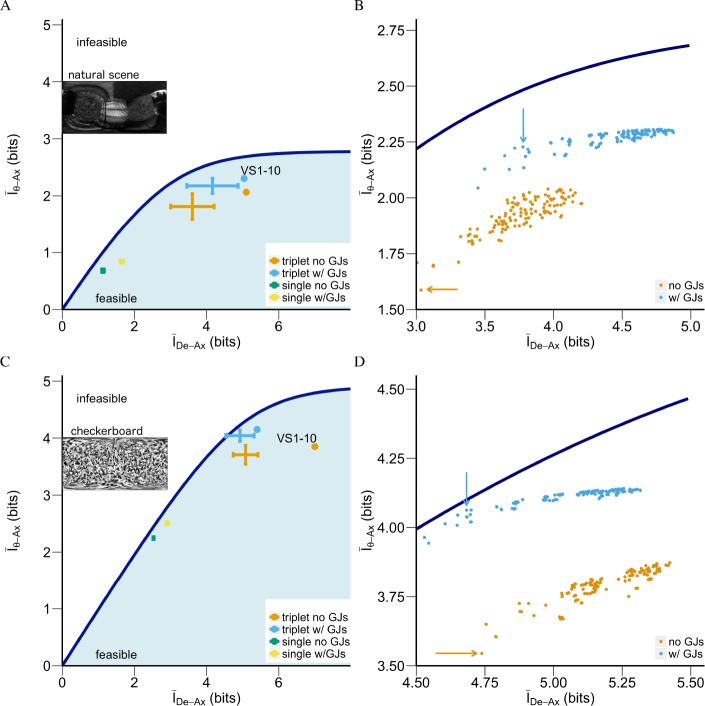
Near-optimal motion representation with GJs. (**A**) Near optimal motion representation for natural stimuli due to GJs by both triplets of VS cells (blue cross) and by the whole VS network (blue dot). The efficiency of the representation for a subpopulation is denoted by a single point in the *I*_*De−Ax*_ − *I*_*θ−Ax*_ plane, which shows how much information (in bits) corresponds to the neural cost and how much information is provided at this cost to represent the axis of rotation. This plane shows the feasible (blue region) and infeasible (white region) separated by the bound $I_{\theta -Ax}^{*}(I_{De-Ax})$, dark blue line (see **Materials and Methods**) for all axes of rotation of natural stimuli. The error bars depicting encoding efficiencies for triplets with/without GJs (blue vs. orange, respectively). Single cells with/without GJs appear in green/yellow squares, respectively (all 20 individual VS cells behaved very similarly to each other). The encoding efficiency of the whole VS network with/without GJs is shown in blue/orange circles. (B) The scatterplot of efficiencies for representations of all 120 triplets (all possible triplets out of the 10 VS cells; the same triplets were used in both sides of the visual system), with/without GJs (blue/orange respectively). The arrows point to VS5-6-7, the triplet connecting downstream to the neck motor center. Note the considerable improvement in efficiency due to GJs for this triplet. (**C**) Similar to (**A**), but for checkerboard stimuli. (**D**) Similar to (**B**), but for checkerboard stimuli.

In Fig 3, we determined that the VS network, especially the VS5-6-7 triplet, shows near optimal encoding of the axis of rotation based on the information bottleneck method [53].

We used two mutual information metrics to investigate this efficiency: the value (relevancy); i.e., the mutual information between the axis of rotation and the axonal voltage, denoted *I*_*θ*−*Ax*_ and the neural cost (complexity); i.e., the mutual information between the dendritic current and the axonal voltage, denoted *I*_*De*−*Ax*_. *I*_*De*−*Ax*_ is the information we have access to and *I*_*θ*−*Ax*_ is the information we would like to know. Namely, *I*_*θ*−*Ax*_ is the amount of information an encoding can provide about the axis of rotation after it encodes *I*_*De*−*Ax*_ bits from the dendritic input. We define *I*_*De*−*Ax*_ as cost because in order to obtain information about *θ*, the axonal voltage needs to build an encoding of the dendritic input. This encoding would not only contain information about *θ*, but also other aspects of the information from the dendritic input. The mutual information between dendrite and axon quantifies how complex this encoding needs to be in order to encode a specific amount of information about θ. Using the information bottleneck method (Tishby, Pereira, & Bialek, 1999), we obtained the so-called information curve: every point of this curve denotes the maximum *I*_*θ*−*Ax*_ (denoted $I_{\theta -Ax}^{*}$) for the respective *I*_*De*−*Ax*_ cost.

To investigate the encoding efficiency, in terms of (*I*_*De*−*Ax*_, *I*_*θ*−*Ax*_), of the encodings for *θ*, by the axonal voltage in the VS network, we generated data following the procedure shown in Fig 1. The dataset contained samples for each axis of rotation, from 0 to 360 in 1° steps. This uniform sampling was recently shown to be behaviorally relevant [29]. Each sample was a combination of input current and output voltage (as in Fig 1C), generated by projecting a randomly selected natural scene (Fig 1A) onto the modeled visual system (Fig 1B, see **Materials and Methods**). Note that the major fluctuation of the input to the dendrites came from the random instantiation of the embedded scenes. Based on these data, Fig 3 plots the encoding efficiency, (*I*_*De*−*Ax*_, *I*_*θ*−*Ax*_), for three VS subpopulations: (i) all individual VS cells; (ii) all 120 VS triplets and (iii) the whole VS network to investigate how GJs changed their respective encoding efficiency.

Fig 3A and 3C show the information curve (in dark blue) for natural and checkerboard stimuli, respectively. Every point on this curve denotes the optimal *I*_*θ*−*Ax*_ with a specific neural cost *I*_*De*−*Ax*_. When we compare a realistic encoding with (*I*_*θ*−*Ax*_, *I*_*De*−*Ax*_) to the information curve, we compare the information it obtains about the stimuli, namely the *I*_*θ*−*Ax*_ with this limit, denoted as $I_{\theta -Ax}^{*}(I_{De-Ax})$. By doing this, we can evaluate if a particular encoding is optimal or not. In addition, note that both curves have a favorable region, the “shoulder”, such that encodings located at this region have the most value for *I*_*θ*−*Ax*_ whereas the ratio of the value to the cost still remains high. Any encoding with a neural cost *I*_*De*−*Ax*_ that is higher than the *I*_*De*−*Ax*_ for the “shoulder” will suffer from a “diminishing return”; i.e., increasing the cost will not gain much on the motion information, *I*_*θ*−*Ax*_. Specifically, we define the shoulder region as the segment where the derivative of ratio between *I*_*θ*−*Ax*_/*I*_*De*−*Ax*_ changes the fastest, i.e., the top 10% in magnitude of the entire information curve. Thus, the shoulder region for natural stimuli has *I*_*De*−*Ax*_ between (3.11, 3.90) bits (Fig 3A) and for checkerboard stimuli has *I*_*De*−*Ax*_ between (5.56, 6.34) bits (Fig 3C). Therefore, when we compare two encodings with respect to the information curve, we first favor the encoding with higher optimality, which is evaluated according to their own respective $I_{\theta -Ax}^{*}(I_{De-Ax})$ from the information curve, then we will prefer the encoding which is closer to this shoulder region because it extracts significant amount of information without suffering the effect from the law of diminishing returns.

Fig 3A and 3C show that all triplets (blue crosses) and the whole VS 1–10 network (blue circles) encoded more information about the stimuli with GJs than the case without GJs (orange cross and orange circle, respectively). In natural stimuli with GJs, the VS 1–10 reached 2.306 (± 0.001)/2.685 = 86% of its respective limit $I_{\theta -Ax}^{*}(I_{De-Ax})$. Interestingly, the mean efficiency of all triplets when the network had GJs reached 87% of their respective limits. For the checkerboard stimuli, these ratios became 4.150 (±0.004)/4.430 = 93% and 94%, respectively. With GJs, they all operated near-optimally. Furthermore, the encoding using triplets was superior to that of the whole VS network, because triplets have lower neural cost (*I*_*De*−*Ax*_ is lower). In addition, the total 120 triplets were divided to a few clusters according to their tuning curve spacing (see S5 Fig). We define the tuning spacing as the maximal angular distance between cells in the triplet. For example, the spacing in VS 1-6-10 triplet is 144° which is ±154° (the RF center for the left and right VS10 cells, respectively) minus ±10° (the RF center for the left and right VS1 cells, respectively). In general, the larger the tuning spacing, the higher the *I*_*De*−*Ax*_. All triplets with tuning spacing of 80° or more form one cluster (shown in purple). The triplets with boundary VS cells (VS1 and VS10) tend to have similar *I*_*De*−*Ax*_ compared to other triplets with narrower tuning spacing but without boundary VS cells. i.e., triplet VS 1-2-4 with tuning spacing of 48° has similar *I*_*De*−*Ax*_ as that of VS 2-3-4 (for which the tuning spacing is 32°) rather that of VS 2-3-5 (for which the tuning spacing is 48°, see the three arrows in S5 Fig). As shown in this figure, all triplets encode similar amount of information in *I*_*θ*−*Ax*_; indicating that those triplets with narrow tuning spacing are therefore preferred for encoding θ because of their moderate *I*_*De*−*Ax*_. Surprisingly, the encoding using single cells did not benefit from GJs as much. They in general represented more information about the axis of rotation, but GJs increased their neural costs which pushed these single cell encodings farther away from their respective limits (Fig 3A and 3C, green and yellow squares at the lower left). It is also worth noting that with GJs or without GJs, all single VS cells were similar in terms of the motion information they represented, as well as their respective neural costs.

Several features of the encoding by the VS 5-6-7 triplet with GJs make it unique. It was the closest to its respective physical limit among all triplets; namely its ${I_{\theta -Ax}/I}_{\theta -Ax}^{*}$ ratio was 2.228 ± (0.004)/2.478 = 90% for the natural stimuli and 4.060 ± (0.003)/4.1 = 99% for the checkerboard stimuli (blue arrows in Fig 3B and 3D, respectively). This is much higher than the ${I_{\theta -Ax}/I}_{\theta -Ax}^{*}$ ratio achieved by the same VS 5-6-7 triplet without GJs: without GJs, the encoding by the VS 5-6-7 triplet only has ${I_{\theta -Ax}/I}_{\theta -Ax}^{*}$ ratio as 1.587 ± (0.003)/2.231 = 71% in natural stimuli and 3.54/4.061 = 87% in checkerboard stimuli (orange arrows in Fig 3B and 3D, respectively). In other words, the difference in optimality with versus without GJs is visible in both Fig 3B and 3D when we compare the blue versus the orange arrows (and their respective points). The most important feature of the encoding by the VS 5-6-7 triplet was that, with GJs, its *I*_*De*−*Ax*_ makes it locates within the shoulder region of the information curve for the natural stimuli (where *I*_*De*−*Ax*_ between (3.11, 3.90) bits, see Fig 3A and 3B), achieving 90% of its respective limit in *I*_*θ*−*Ax*_. This shows that for natural statistics, this triplet encoding achieved a nearly optimized return in value *I*_*θ*−*Ax*_ through a modest *I*_*De*−*Ax*_ cost. Therefore, with GJs, the VS 5-6-7 triplet was optimized to encode motion in the natural environment.

### Efficient encoding by the VS 5-6-7 triplet is robust regardless of signal-to-noise ratio (SNR)

**Fig 4:**
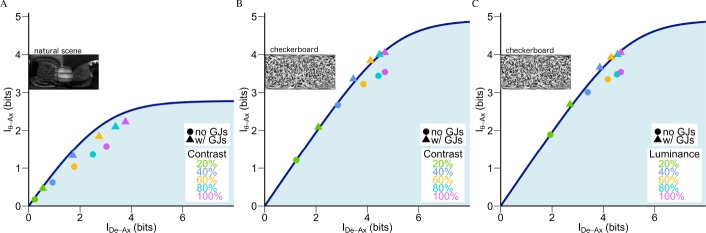
With GJs, the near-optimality encoding by the VS 5-6-7 triplet is robust over a wide range of signal-to-noise ratios. (**A**) The information curve and the encoding efficiency of the axis of rotation by the VS 5-6-7 triplet in the *I*_*De*−*Ax*_ − *I*_*θ*−*Ax*_ plane to varying contrast levels of natural stimuli (contrast is coded by colors as shown in inset). The cases with GJs are represented by triangles and without GJs by circles. The blue curve is the same as in Fig 3. Note that for the case represented by the orange triangle (60% contrast with GJs) more information *I*_*θ*−*Ax*_ is extracted about motion and is closer to the information curve as compared to the orange, cyan and purple circles (representing 60%, 80% and 100% contrast without GJs, respectively). (**B**) As in (**A**), but using checkerboard stimuli. (**C**) As in (**B**) but with different luminance levels.

Reducing the SNR of the stimuli can reduce the information transmission of the photoreceptor [54]. Therefore, changing the SNR will also change the encoding efficiency of motion. To investigate whether the near-optimality of the encoding by the VS 5-6-7 triplet shown in Fig 3 was robust regardless of the SNR we performed the above analysis on separately generated datasets by changing the luminance (linearly changing SNR) and contrast of the checkerboard stimuli and the contrast (quadratically changing SNR) of the natural stimuli (see **Materials and Methods**). Fig 4 depicts how the near optimality varies with changes in the SNR by the VS 5-6-7 triplet.

As shown in Fig 4A–4C, GJs enable motion encoding by the VS 5-6-7 triplet to operate near its respective physical limits, regardless of the wide variation in signal-to-noise ratios. Compared to the same representation without GJs (shown in circles), having GJs emerged as especially beneficial for low SNR scenarios. For example, when the contrast was set at 60% for the natural stimulus, the encoding by the VS 5-6-7 triplet reached 1.851 ± 0.002 bits about the axis of rotation (orange triangle in Fig 4A). Given that the neural cost for this case was *I*_*De*−*Ax*_ = 2.74 ± 0.002 bits, the information curve in Fig 4A shows that the respective motion information limit was at $I_{\theta -Ax}^{*}$ = 2.09 bits. Hence, it achieved ($I_{\theta -Ax}/I_{\theta -Ax}^{*}$) 1.851 ± (0.002)/2.090 = 88% of this physical limit. This encoding efficiency not only outperformed the scenario where the GJs were absent but the contrast stayed the same (orange circle), but also outperformed the scenarios which were without GJs but the contrast was higher (higher SNR). In particular, when the stimulus had a 100% contrast, without GJs, the encoding by the VS 5-6-7 triplet could only extract 1.587 ± 0.003 bits about the axis of rotation (top left-most purple circle in Fig 4A). This value was lower than the 1.851 ± 0.002 bits that was extracted at a lower contrast when GJs were present. Furthermore, this 1.587 ± 0.003 bit value was suboptimal (its $I_{\theta -Ax}/I_{\theta -Ax}^{*}$ was only 71%). This finding indicates that GJs may be better at improving the encoding efficiency of motion than that from enhancing the stimulus contrast (which is a typical strategy for improving SNR).

For the checkerboard stimuli (Fig 4B and 4C), GJs had a range of effects. At a low SNR (when the luminance and contrast were low), the encoding by the VS 5-6-7 triplets added additional bits to the cost (the x-axis) with GJs but also extracted more motion information (the y-axis; e.g., compare green circle to green triangle in Fig 4B). For example, with a 20% luminance (green circle and triangle in Fig 4C), GJs added a 0.8 bit cost (the *I*_*θ*−*Ax*_ axis) to the encoding by the VS 5-6-7 triplet but resulted in a 0.7 additional bit improvement in *I*_*θ*−*Ax*_ (the green triangle is above the small green circle). At a high SNR (when the luminance and contrast are both high), the encoding by the VS 5-6-7 triplet extracted more bits about the axis of rotation, but without a significant change in the cost *I*_*De*−*Ax*_. For example, for 100% luminance, the encoding by VS 5-6-7 when connected by GJs achieved 0.46 more bits in *I*_*θ*−*Ax*_ without changing *I*_*De*−*Ax*_ (compare the purple circle to the purple triangle in Fig 4C).

### With GJs, the VS 5-6-7 triplet successfully encodes motion information critical for behavior

**Fig 5:**
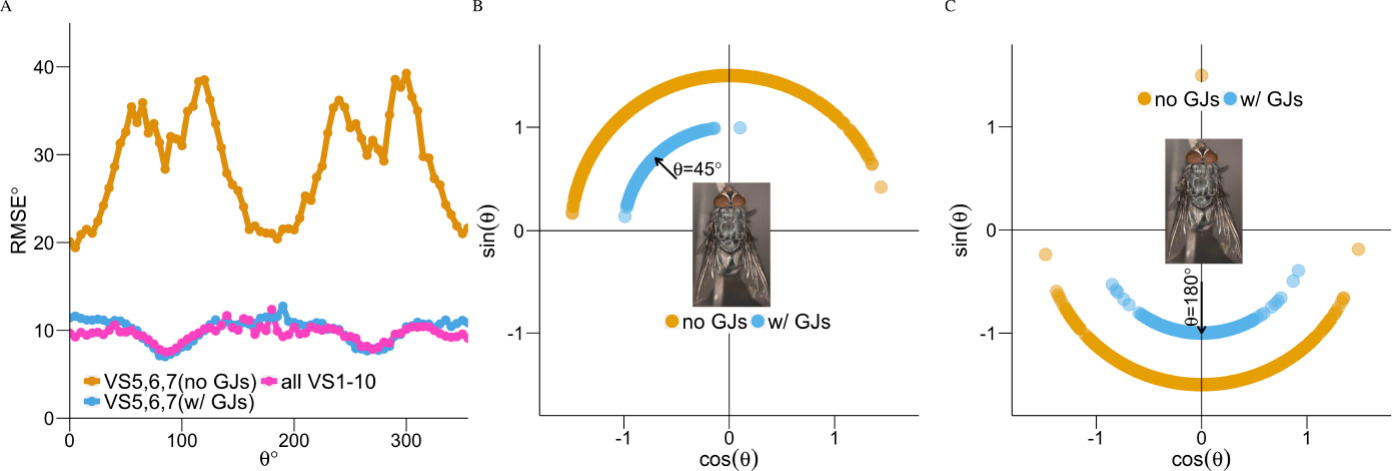
GJs are particularly important for the fly's survival when encoding is based on the VS 5-6-7 triplet. (**A**) RMSE for estimating axes of rotation (1600 samples, each in 5° steps) based on the encoding by the VS 5-6-7 triplet with GJs (blue) and without GJs (orange). The RMSE using all VS 1–10 cells with GJs is shown in magenta. (**B**) The variability of the estimated axis of rotation for the case of *θ* = 45° with (blue, with radius 1) and without (orange, with radius 1.5) GJs. Note that with GJs, the error falls within the same quadrant whereas without GJs the error is almost 180°. This means that without GJs, the fly cannot encode pitch axis correctly. Since the VS 5-6-7 triplet is connected downstream to the fly motor system, GJs are essential for the fly’s behavior, e.g., avoiding swats (see text). (**C**) Similar to (**B**), but for the *θ* = 180° stimulus.

To better understand the importance of this efficient encoding by the VS 5-6-7 triplet with GJs (Fig 3B and 3D), we investigated its impact on behavior based on a separate test dataset made up of 1600 samples for each axis of rotation in 5° steps for natural stimuli. We tested how well the encoding by the VS 5-6-7 triplet performed in estimating the axis of rotation *θ*. We evaluated the performance of the VS 5-6-7 triplet (with and without GJs) by calculating the root mean square error (RMSE) between the estimated axis of rotation (*θ*^*est*^) and the specific target *θ* (Fig 5).

Fig 5A shows that as expected with GJs, the encoding by the VS 5-6-7 triplet is significantly better in estimating the axis of rotation compared to that without GJs. Without GJs (Fig 5A, orange trace), the estimation error for each individual axis of rotation was modulated by the distance from the center of the RF for VS5, VS6 and VS7 (±74°, ±90°, ±106° respectively, Table 1). Thus, without GJs the errors were large (up to 40° for *θ* = 90°) for the axes of rotation that were close to the center of the respective RF (the large peaks in Fig 5A), because the motion input near their axis of rotation was small (S1 Fig). The blue and magenta traces in Fig 5A show that with GJs all errors were within 10°. Fig 5A also shows that the encoding by the VS 5-6-7 (blue trace) was as good as when the whole VS network was used (magenta trace).

Fig 5B and 5C depict the behavioral superiority of GJs. We compared the variability in estimation using the encoding by the VS 5-6-7 triplet for two different target stimuli, *θ* = 45° and *θ* = 180°. The case of *θ* = 45° corresponds to combining a roll (turn clockwise/counterclockwise) and a pitch (tilt up/down), as when the fly performs a banked turn whereas *θ* = 180° corresponds to a clockwise roll. When the target *θ* = 45°, without GJs the variability in estimation using the encoding by the VS 5-6-7 triplet spanned almost 180° and was asymmetrical around 45°; the magnitude of the clockwise error exceeded 90° (Fig 5B, orange). This indicates that without GJs, the encoding by the VS 5-6-7 triplet cannot correctly distinguish the optic flow corresponding to the fly tilting upwards or downwards, thus it may confuse the 45° with -45°. In the presence of GJs (Fig 5B, blue) however, the error was within the same quadrant of the target stimulus (the blue band around *θ* = 45°). When *θ* = 180°, the improvement in estimation due to GJs was mainly manifested in the reduction of the standard deviation of the estimation error (Fig 5C). Thus, although having GJs can reduce the standard deviation of the estimation error, its main advantage is to provide critical behaviorally related information so that the upward and downward rotation can be encoded correctly. S2 Fig shows that in the case of checkerboard stimuli, GJs can in addition lead to a hyperacuity level of discrimination [55].

Thus overall, having GJs improves the encoding by the VS 5-6-7 triplet for those optic flow patterns which are resulted from common maneuvers the fly perform, such as banked turns. This underscores the functionality of GJs in the VS network in enabling the fly’s behavior.

## Discussion

To successfully apply information theory to analyze a biological system, it is critical to know which information is relevant. Here we focused on a physiologically realistic model of the VS network in the fly visual system [18], which is known to encode motion information (Fig 1 and [23]). The analysis showed that within the 10 ms of stimulus onset, with axonal GJs, the VS system could efficiently encode the axis of rotation, *θ* (Fig 3A and 3C). In particular, efficient encoding was also achieved by the VS 5-6-7 triplet (Fig 3A and 3C) which is the only known output of this network. Although the entire VS network operates near-optimally, the encoding by the VS 5-6-7 triplet emerged as superior to that of the whole network, and was robust regardless of the signal to noise ratio (Fig 4). In addition, the efficiency by the VS 5-6-7 triplet was shown to correspond to the favorable region in the information curve (Fig 3) where the gain of motion information, *I*_*θ*−*Ax*_, is maximized with modest *I*_*De*−*Ax*_ cost (Fig 3A and 3B). In addition to identifying the emergence of efficient encoding of the axis of rotation, we also assessed quantitatively the extent to which GJs in the VS network are critical to successful visually guided behavior. This constitutes a step forward from previous experiments [17] which only quanlitatively suggested that GJs might contribute to reducing the fluctuations in the pitch axis (tilt up/down). Here we analyzed the uncertainty distribution of the estimated axis of rotation, and showed that the VS 5-6-7 triplet can encode all axes of rotation uniformly well in the presence of GJs (Fig 5B and 5C). However, without GJs, the encoding by the VS 5-6-7 triplet yield errors of up to 90° for those rotations including a pitch component (tilt up/down). Thus, this result predicts that without GJs, maneuvers involving a substantial pitch rotation component should be error-prone. This is detrimental to many free flight maneuvers. For example, the banked turn, which is usually initiated when escaping predators, always involves a pitch rotation (tilt of the head up or down) along with a roll rotation [29, 51]. It is critical for the fly's survival to perform these maneuvers accurately, which points to the need to have GJs in the corresponding VS network. Hence, not only that GJs enable efficient encoding of motion to emerge in the VS network but, in addition, that this efficient encoding is essential to subsequent visual guided behaviors.

### Comparison to related studies on the VS network

Only a few studies [56, 57] have attempted to use a probabilistic approach to investigate population coding of motion in the VS network. These studies have focused exclusively on how well the axonal voltage, with/without GJs, could be used to estimate the axis of rotation. Although this estimation can be generalized to a wide spectrum of tasks [58]*)*, it is still not as parsimonious as the measure of motion information (in bits) that we provided in this study. Our measure of motion information quantifies the absolute amount of information available to perform all possible tasks. Unlike the previous approach (which hypothesized that reading out from the whole VS network is optimal in decoding), we used the information bottleneck method [53] that enabled us to evaluate the physical limit of encoding efficiency, which is solely determined by the statistical structure of the visual input itself. This meant we could assess how well the system performs with respect to its respective physical limit without making any assumptions. We were able quantitatively show that the VS 5-6-7 triplet outperforms the whole VS network in terms of encoding efficiency (Fig 3). We further shed light on the behavioral superiority of having GJs, by inspecting which errors they could correct on both estimation and discrimination tasks (Fig 5 and S3 Fig), which was not investigated in previous studies.

### GJs in the VS network provide a novel mechanism for efficient encoding in the fly visual system

The blowfly visual system is a classical model for studies on efficient encoding of motion. Most previous studies have been conducted on the H1 neuron, a LPTC neuron selective for horizontal inward motion, but without known direct connections to the VS network [40, 59, 60]. It was shown that the H1 neuron efficiently uses of its spiking capacity to transmit information and is highly adaptive for natural stimuli. Its encoding efficiency is close to the its physical limit [40] as we have shown for the VS system. These works claimed that a single neuron can generate efficient encoding of its input. We complement this view on the efficient encoding of motion by showing that the specific synaptic connectivity may also serve as a candidate mechanism for efficient encoding in a population-coding paradigm. In this perspective, our work broadens the applicability of a previously known computational principle to describe how circuitry features supports efficient encoding of sensory stimuli

The VS neurons consist of an elaborated dendritic tree and an axon that is connected via GJs to nearby axons. Therefore, the VS cells in our model consist of a separate dendritic and axonal compartment (Fig 1). Importantly, this implies that GJs facilitate information transmission among axons while maintaining the information received by the dendrites intact. Thus, the efficient coding mechanism suggested in our work is fundamentally different from those observed in “point neuron” models, which can only interpret improving the mutual information between input and output as increasing of energy expenditure on single cells [61, 62]. To the best of our knowledge, this study is the first to demonstrate that the efficient encoding of sensory stimuli can be induced by axonal GJs.

### Future work

There are two natural extensions of the present study. The first is to extend the single cell model to include the elaborate dendritic morphology of real VS cells. It is known that local motion detectors (LMD) units impinge on distal small dendritic branchlets of VS cells [48]. Given that LMDs function on time scales that are about ten times slower than those of the membrane time constant of VS cells [18], the input to the dendrites is likely to be sparse. This may impact the encoding strategy implemented by the VS system. This leads to the intriguing question of whether and how the dendritic structure might influence the near optimality of the encoding efficiency as found in the present study. We hypothesize that including dendrites would illustrate how the “roll” motion (clockwise/counterclockwise turn) is encoded, since previous experiments have shown that the dendritic morphology can reduce fluctuations in the vertical direction [63]. Furthermore, the biological VS network receives input from both the compound eyes and the ocelli [64], whereas our simulation only considered how efficiently the VS network represents its input from the compound eyes. We predict that additional information from the ocelli should improve the motion representation of the VS network [64].

The second extension would be to investigate why the 10 ms following the stimulus onset is the relevant timescale for the efficient encoding of motion. As shown in S2A Fig, most motion information is obtained by the VS5-6-7 triplet within the first 10 ms. Considering that a change in rotation in response to a visual cue takes ~30–40 ms [29], it remains unclear why the saturation of motion information encoding should take place in the first 10 ms. In other words, how does the information available in the first 10 ms act predictively for the behavioral state of the fly at later times? Addressing this issue could shed light on whether the efficient encoding of motion in the fly visual system matches the efficient encoding of predictive information, a known principle that governs population coding in the retina [65].

Given the presence of gap junctions in many neuronal systems, we believe that our study will encourage the exploration of GJs as a common mechanism for improving information transmission in these systems. Initial evidence suggests that GJs improve information transmission in both the periphery (retina, shown in [7]) as well as in neocortical L2/3 inhibitory interneuron networks [66]. The discussion in [19] supports the idea that low-level motion detection follows a common circuit design in both fly and mammalian motion vision. However, the extent to which this commonality also holds throughout the central nervous system has yet to be studied.

## Materials and methods

### The simplified VS model

The modeled VS network used in the present work (Fig 1) was first introduced in [18]. In this model, each VS cell is represented by a dendritic compartment connected, axially, to an axonal compartment. This model was validated in [18] where they used genetic algorithm to match the axial and membrane resistances of each VS cell, such that steady-state potentials at the dendritic root resulting from current injections into a certain VS cell match the experimental data. As a second constraint for the parameter fit the VS cells had to approximate an input resistance of about 4MΩ. This model was then used in several follow-up studies e.g., [67] and [56].

### Simulation of the model VS network

We used the model VS network (Fig 1) first introduced in [16, 18] in this study. This model uses two compartments to describe an individual VS cell. It defines the receptive field (RF) of these dendritic compartments as 2-D Gaussian with *σ*_*azimuth*_ = 15° and *σ*_*elevation*_ = 60°, tiling along the anterior-posterior axis (Fig 1). The neighboring axonal compartments of different VS cells are connected by GJs, whereas VS1 and VS10 are connected by inhibitory chemical synapses. In our simulation, all conductance magnitudes and the inhibition magnitude were set using the same method as in [16, 18, 56]. We only varied the magnitude of the GJ conductance between 0 and 1 μS. We chose 1 μS because this is the value used previously [18], which confirmed that with this GJ, this reduced model displayed behavior similar to a realistic VS cell.

In every simulation, we first generated the “cage” (Fig 1) either by randomly selecting six images from the van Hateren dataset [51] or by randomly generating six checkerboard images. Then, we rotated this cage at a specific axis of rotation (500°/s based on experimental findings [68, 69, 70]. This yielded the optic flow pattern which we then fed into the 5000 local motion detectors (LMD) [52]. Each LMD comprised two subunits that differed by 2° in elevation. They were randomly distributed in the sphere mimicking the visual range of the fly. Each VS dendrite used the output of LMDs, which fell into its respective RF to generate the input current to the model VS network. The temporal average of the resulted axonal voltage $V_{Ax}=\frac{1}{T}\int V_{Ax}(t)dt$ (t = 10 ms) was used for our subsequent analysis.

### Model joint distributions as Gaussian copula

Similar to [56], we also used the Gaussian copula to model high dimensional joint distributions; i.e., the joint distribution between the current and the axis of rotation *P*(***curr***,***θ***) and the joint distribution *P*(***V***_***Ax***_) for the representation of subpopulation VS cells. For an N-dimension random variable: (*R*_1_,…,*R*_*N*_), the copula *C* (Sklar's theorem, [71]*)* is defined as follows:

$$F\left(R_{1},\ldots ,R_{N}\right)=C\left(F_{1}\left(R_{1}\right),\ldots ,F_{N}\left(R_{N}\right)\right)$$ (1)

where *F*_*i*_(*R*_*i*_) = ∫ … ∫ *F*(*R*_1_,…,*R*_*N*_)*dR*_1_ … *dR*_*i*−1_*dR*_*i*+1_*dR*_*N*_ is the marginal cumulative distribution of the variate *R*_*i*_. With a new variable *U*_*i*_ = *F*_*i*_(*R*_*i*_), the Gaussian copula is a parameterized copula defined by the correlation matrix *Σ* in which its diagonal entries are Σ_*ii*_ = 1, and its off-diagonal entries are ∑_*ij*_ = *corr* (Φ^−1^(*U*_*i*_), Φ^−1^(*U*_*j*_)). The Gaussian copula then has the following form:

$$C_{Gauss}^{\Sigma }\left(u\right)=\Phi_{\Sigma }\left(\Phi^{-1}\left(U_{1}\right),\ldots ,\Phi^{-1}\left(U_{N}\right)\right)$$ (2)

in which the Φ^−1^ is the inverse cumulative function of the standard normal distribution, and Φ_∑_ is the cumulative distribution function of the multi-dimensional Gaussian distribution with a covariance matrix defined by *Σ*. Correspondingly, for a given vector (*R*_1_,…,*R*_*N*_), the copula density has the following form:

$$c_{Gauss}^{\Sigma }=\frac{1}{\det\left(\Sigma \right)}\exp\left[w^{t}\left(\Sigma^{-1}-I\right)w\right]$$ (3)

where ***w*** = (Φ^−1^(*U*_1_),…,Φ^−1^(*U*_*N*_)).

Therefore, for a given (*R*_1_,…,*R*_*N*_) the density is defined as follows (by combining the Gaussian copula and the marginal distribution functions):

$$P\left(R_{1},\ldots ,R_{N}\right)=c_{Gauss}^{\Sigma }\left(U_{1},\ldots ,U_{N}\right)\prod_{i=1}^{N}\frac{d}{dR_{i}}F_{i}\left(R_{i}\right)$$ (4)

where the $\frac{d}{dR_{i}}F_{i}(R_{i})$ denotes the marginal probability density for each variate *R*_*i*_.

### Goodness of fit for Gaussian copula between dendritic input and the axis of rotation

Fig 4 in [56] showed that the Gaussian copula can capture most of the dependence structure in the joint distribution of VS subpopulation axonal responses. Here, we present the goodness of fit test for the Gaussian copula of the joint distribution *P*(***curr***,***θ***) combining the dendritic input ***curr*** and the axis of rotation **θ**. Initially, this task looks prohibitive because of the high dimensionality of the dendritic currents (dimension d = 20). However, this 20-dimensional current input has only two principal axes (S6A and S6C Fig). Hence, we only need to validate a 4-dimensional Gaussian copula composed of the two principal axes from the current and the two-dimensional transformation (*cosθ*,*sinθ*). S6B and S6D Fig show that the Gaussian copula indeed captures most of the dependence structure of the joint distribution *P*(***curr***,***θ***).

### Mutual information estimation

We used the k-nearest neighbor approach described in [72] to obtain the mutual information measures *I*(***curr***,***V***_***Ax***_) and *I*(***V***_***Ax***_,***θ***). Here, the mutual information *I*(***X***,***Y***) between two distributions, **X** and **Y**, were evaluated as the derivative of a complete gamma function (for details, see [72]) as follows:

$$I\left(X,Y\right)=\psi \left(k\right)-\langle \psi \left(n_{x}+1\right)+\psi \left(n_{y}+1\right)\rangle +\psi \left(N\right)$$ (5)

k is the number of nearest neighbors (we set k = 11, because this is the value at which we observed the estimation to converge [72]. *n*_*x*_ and *n*_*y*_ are determined based on k.

When we report the *I*(***curr***,***V***_***Ax***_) and *I*(***V***_***Ax***_,***θ***), we report a cross-validated mean of the above computation. Namely, we divided a dataset of 360,000 samples (1000 samples for each axis of rotation) into five subsets, and report the mean mutual information estimation. We omitted the standard deviations when we plotted Fig 3, Fig 4 because of their small magnitudes (<0.01).

### Efficient encoding of motion based on the information bottleneck method

Not all information transmitted from the photoreceptors are about motion, hence we can build a compressed representation of motion if we know which information we need to keep (relevant information) and which are not from the input. The information bottleneck method [48] treats *V*_*Ax*_ as the representation subject to be optimized (the “bottleneck”). The representation *V*_*Ax*_ has a value, i.e., how much motion information is represented (relevancy) and a neural cost (complexity), i.e., how much information is obtained from its input. Mathematically, we can define the relevant information as *I*_*θ*−*Ax*_ = *I*(***θ***,***V***_***Ax***_), the mutual information between any representations and the axis of rotation ***θ*** and the neural cost as the mutual information between the joint axonal voltage and the dendritic current: *I*_*De*−*Ax*_ = *I*(***curr***,***V***_***Ax***_). It is also the complexity of the “bottleneck” ***V***_***Ax***_. The optimally efficient encoding (“bottleneck”) minimizes the following variational principle:

$$L_{IB}\left[P\left(V_{\mathrm{Ax}}|\mathrm{curr}\right);\beta \right]=I_{De-Ax}-\beta I_{\theta -Ax}$$ (6)

where *β* is the trade-off parameter between saving the neural cost; i.e., reducing the complexity *I*_*De*−*Ax*_ and increasing the value; i.e., increasing the relevant information *I*_*θ*−*Ax*_.

In general, this problem is difficult. However, given that the combined distribution of the current inputs is a Gaussian copula, we can obtain an analytic solution for this particular VS network, following the meta-Gaussian information bottleneck framework [73, 74]. Based on this framework, we can use the left eigenvector and left eigenvalues of the matrix $M=\Sigma_{De|\theta }\Sigma_{De}^{-1}$ to determine the optimal representation.

Therefore, for the whole range of *I*_*De*−*Ax*_, we adapted the Eq 11 from to calculate their respective optimal relevant information *I*_*θ*−*Ax*_. We thus obtained:

$$I_{De-Ax}=I_{\theta -Ax}+\frac{n_{I}}{2}log\left(\prod_{i=1}^{n_{I}}{\left(1-\lambda_{i}\right)}^{\frac{1}{n_{I}}}+exp\left(\frac{2I_{De-Ax}}{n_{I}}\right)\prod_{i=1}^{n_{I}}\lambda_{i}{}^{\frac{1}{n_{I}}}\right)$$ (7)

where *λ*_*i*_ are the left eigenvalues of M in ascending order. *n*_*I*_ is the cutoff number of eigenvalues that we used to estimate the information curve. Starting at *I*_*De*−*Ax*_ = *I*_*θ*−*Ax*_ = 0, we estimated the information curve composed of several segments with an increasing number of eigenvalues.

### Manipulation of the stimulus

#### Luminance

To change the luminance of an image, we scaled the whole image intensity to the desired level; e.g., 0.2.

#### Contrast

To change the contrast of an image, we subtracted the mean luminance and added it back after we scaled the residue pixel intensity to the desired contrast level; e.g., 60%.

The difference between changing the luminance and changing the contrast is that changing the luminance changes the mean whereas changing the contrast does not.

### Estimation of the axis of rotation

Similar to the method in [56], we report the estimation performance based on a dataset of 1600 samples that were embedded within the natural stimuli between 0° and 360° in 5° steps. For a given axonal voltage vector *R* in the test set, we estimated its corresponding rotation axis as the expectation *θ*^*est*^ given *R*. We first represented this with the rotation vector *s*(*θ*) = (*cosθ*,*sinθ*), and then computed the expectation of this vector with *s*^*est*^ = *E*(*s*|*R*) = ∫ *s*(*θ*)*P*(*θ*|*R*)*dθ* to obtain *θ*^*est*^. Following Eq 4, we then obtained *P*(*θ*|*R*) by combining the fit copula and the marginal distributions of individual axonal responses. For the estimation performance, we used the Root Mean Square Error (RMSE) between the estimated *θ*^*est*^ and the real *θ*, averaging over all 1600 samples.

$$RMSE\left(\theta \right)=\sqrt{\frac{1}{1600}\Sigma_{\theta }{\left(\theta^{est}-\theta \right)}^{2}}.$$ (8)

### Likelihood ratio

We used the checkerboard stimuli in the discrimination task. Here, the test dataset had 1600 samples for the axis of rotation between 0° and 360° in 1° steps (as opposed to the coarser 5° steps for the natural stimuli). For the task, we used the likelihood ratio between two hypotheses: *θ* and *θ*′, with respect to the response vector *R* to determine which stimuli corresponded to R. The ratio was defined as the likelihood that a specified response vector corresponded to *θ* compared to the likelihood that this response vector corresponded to *θ*′.

$$\Lambda \left(R\right)=\frac{L(\theta |R)}{L(\theta^{\prime }|R)}$$ (9)

Here, the likelihood L(θ|R)=P(R|θ).

In the discrimination task, the likelihood ratio determines that *R* corresponded to *θ* if Λ(*R*) > 1, and to *θ*′ otherwise.

### Discriminability

We computed the discriminability *d*′ according to [41]. The higher the discriminability, the higher the success rate in discriminating between two alternative hypotheses. We estimated *d*′ based on the probability of correct discrimination *P*_*c*_ by the equation *d*′ = 2Φ^−1^(*P*_*c*_). Here, $\Phi (x)=\frac{1}{\sqrt{2\pi }}\int_{-\infty }^{x}dz\;exp(-\frac{z^{2}}{2})$, as discussed in [41]. We followed this method to first evaluate the probability of correct discrimination *P*_*c*_, and then to obtain *d*′. In our case, *P*_*c*_ is the mean probability of correctly discriminating whether the response vector *R* corresponds to *θ* against the alternatives of both *θ* + Δ*θ* and *θ* − Δ*θ*, i.e., *P*_*c*_ = Σ_*R*_*P*(*R*|*θ*)*H* (*P*(*R*|*θ*) − *P* (*R*|*θ* + Δ*θ*)) + Σ_*R*_*P*(*R*|*θ*)*H*(*P*(*R*|*θ*) − *P*(*R*|*θ* − Δ*θ*)). The *H* (∙) is the Heaviside step function indicating that only the correctly discriminated samples were included in obtaining *P*_*c*_, which was evaluated over all 1600 samples.

## Supporting information

S1 Fig Optic flow of rotation in the counterclockwise direction around the axis θ (black dashed line). Note that this rotation yields no motion at the axis itself. The further away the respective azimuth degree is from the axis θ (up to 90°), the greater the rotation.
(TIF)

S2 Fig (A) The information about the axis of rotation encoded by the axonal voltages of the VS 5-6-7 triplet with the integration window extending from 10 ms to 50 ms, with (in blue) and without (in orange) GJs, respectively. (B) The colored bars show the information about the axis of rotation encoded by the axonal voltages of the VS 5-6-7 triplet for GJs from 0 to 1 μS in the VS network. Its upper limit appears as the horizontal line; i.e., the amount of information about the axis of rotation available at the dendritic current of the VS network.
(TIF)

S3 Fig Emergence of hyperacuity and improvement in discrimination with GJs for the representation by VS 5-6-7 triplet. **(A**) The discriminability, d’, between θ and θ' (θ—θ’ = Δθ) for all axes of rotation as a function of Δθ, with (yellow) and without (blue) GJs. Error bars indicate one standard deviation. Note that only the blue curve intersects the hyperacuity region whereas the orange curve does not. (**B**) The uncertainty distribution density for θ = 0° (blue histogram) and θ' = 2° (pink histogram) without GJs. The dashed line represents the decision rule, (θ + θ')/2. When a stimulus falls to the left of the dashed line, it belongs to the red distribution; otherwise, to the green distribution. The 85° overlap indicates that this decision rule has a 57% likelihood of being correct (see text). (**C**) Similar to **(B)**, but with GJs. In this case, the (θ + θ')/2 decision rule has a 70% likelihood of being correct, corresponding to the 60% overlap between the two histograms.
(TIF)

S4 Fig Both smoothing (reducing trial-to-trial variability) and improving correlation contributes to better encoding of axis of rotation. (A) Joint axonal voltage response of VS5 versus VS6 in the absence of GJs. A total of 1000 samples for both *θ* = 0° (green) and for *θ* = 60° (red) in response to natural stimuli are shown (see **Materials and Methods**). Their 95% confidence ellipses are shown in black. (B) Shuffled joint axonal voltages of VS5 and VS6 (95% confidence ellipses shown in black); (C) As in (A) but with GJs = 1 μS. (D) Joint axonal voltages for VS5-6-7 of the left compound eye without GJs for six different axes of rotation (indicated by respective colors). (E) Shuffled joint axonal voltages of VS5-6-7 with the same color code as in (D). (F) Joint axonal voltages for VS5-6-7 with GJs = 1 μS. Shuffled joint axonal voltages of VS 5-6-7 (with GJs) still show capability to cluster different axes of rotations (comparing (E) to (D)) but it is inferior to the case without shuffling (comparing (F) to (E)).
(TIF)

S5 Fig Encodings by triplets of VS neurons is divided to clusters according to their tuning spacing. Encoding of triplets for natural stimuli (with GJs), color coded according to the triplet tuning spacing (see text). Note that the e.g., the cluster in red contains both triplets with spacing of 32° as well as triplets with spacing 48° and contain VS1 or VS10. Arrows are pointing to VS 2-3-4, VS1-2-4 and VS 2-3-5, respectively, showing that triplet with boundary VS cells (VS1-2-4 with spacing of 48°) clusters together with VS2-3-4 (with spacing of 32°) rather than with VS 2-3-5 (the cluster in green with spacing of 48°).
(TIF)

S6 Fig Goodness of fit for the Gaussian copula combining dendrite input and stimuli θ. (A) The ten most significant principal components from the currents and the percentages of variance that they explain individually based on the natural stimuli. Note that 90% of the variance can be explained with the two most significant principal components. (B) The quantile-quantile plot the with *P*(***curr***,***θ***), where the current is represented by its two most significant principal components, and ***θ*** is represented as (*cosθ*,*sinθ*). The points are the quantile values of the empirical copula (x-axis) against the fitted Gaussian copula (y-axis) for 10,000 equally spaced points of the form (0.1 m, 0.1 n, 0.1 p, 0.1 q) with 1 ≤ m, n, p, q ≤ 10. We obtained these values based on 360,000 samples, (1000 samples for each individual axis of rotation between 0° and 360°). (C) Similar to (A) but with the checkerboard stimuli. Note that the first two most significant principal components explained all variance. (D) Similar to (B) but with the checkerboard stimuli.
(TIF)

S1 Text With GJs, encoding by the VS 5-6-7 triplet shows hyperacuity level discrimination. (DOCX)
