# Supplementary figures and images for: Efficient encoding of motion is mediated by gap junctions in the fly visual system
Source: PLoS Comput Biol. 2017 Dec 4;13(12):e1005846. doi: 10.1371/journal.pcbi.1005846 (PMC5730180; doi:10.1371/journal.pcbi.1005846)

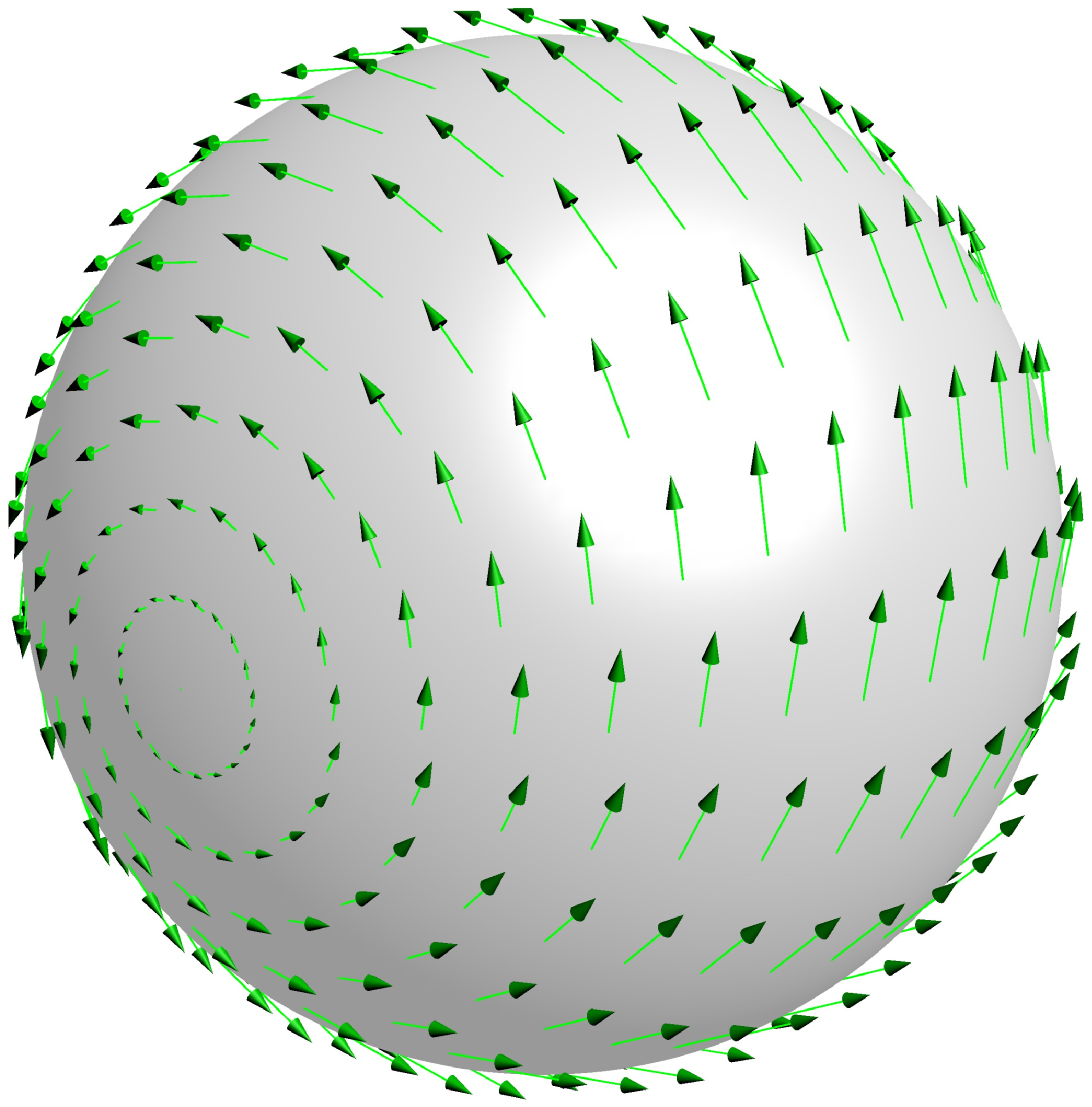

Supplement: S1 Fig — Note that this rotation yields no motion at the axis itself. The further away the respective azimuth degree is from the axis θ (up to 90°), the greater the rotation. (TIF) [file pcbi.1005846.s001.tif]

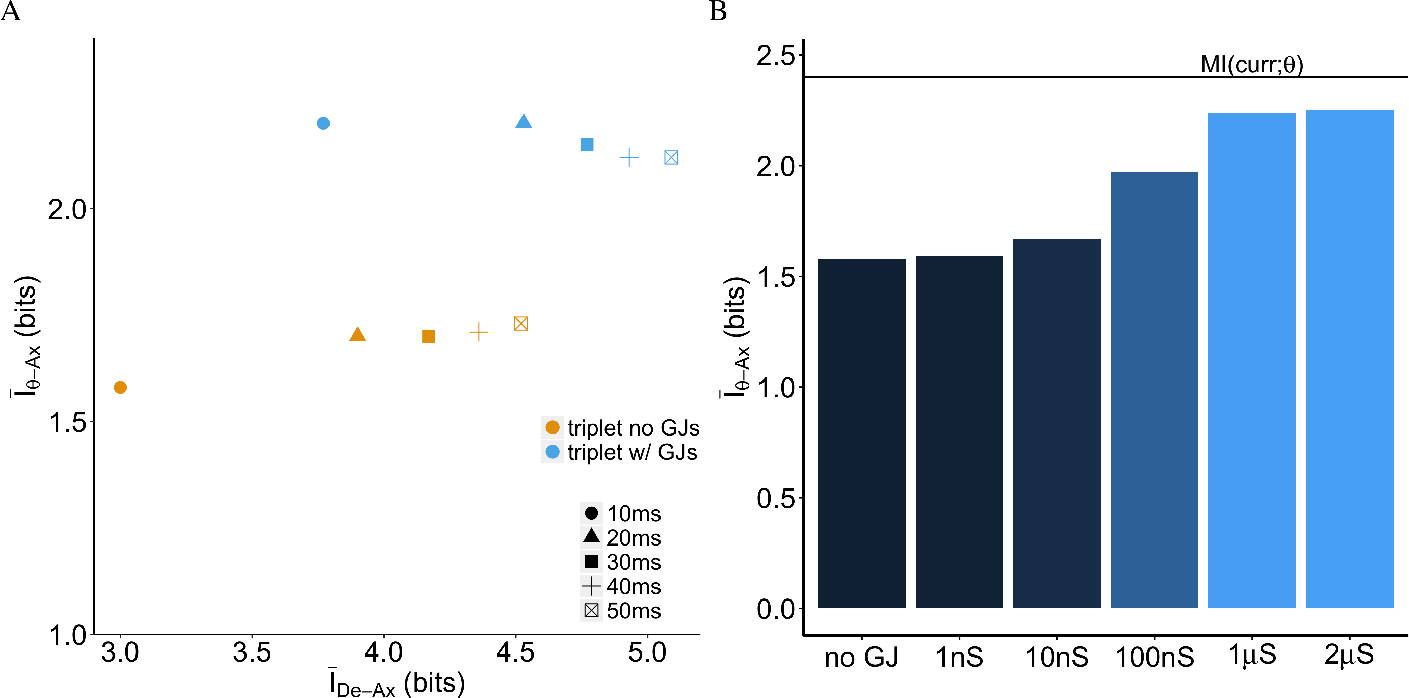

Supplement: S2 Fig — (A) The information about the axis of rotation encoded by the axonal voltages of the VS 5-6-7 triplet with the integration window extending from 10 ms to 50 ms, with (in blue) and without (in orange) GJs, respectively. (B) The colored bars show the information about the axis of rotation encoded by the axonal voltages of the VS 5-6-7 triplet for GJs from 0 to 1 μS in the VS network. Its upper limit appears as the horizontal line; i.e., the amount of information about the axis of rotation available at the dendritic current of the VS network. (TIF) [file pcbi.1005846.s002.tif]

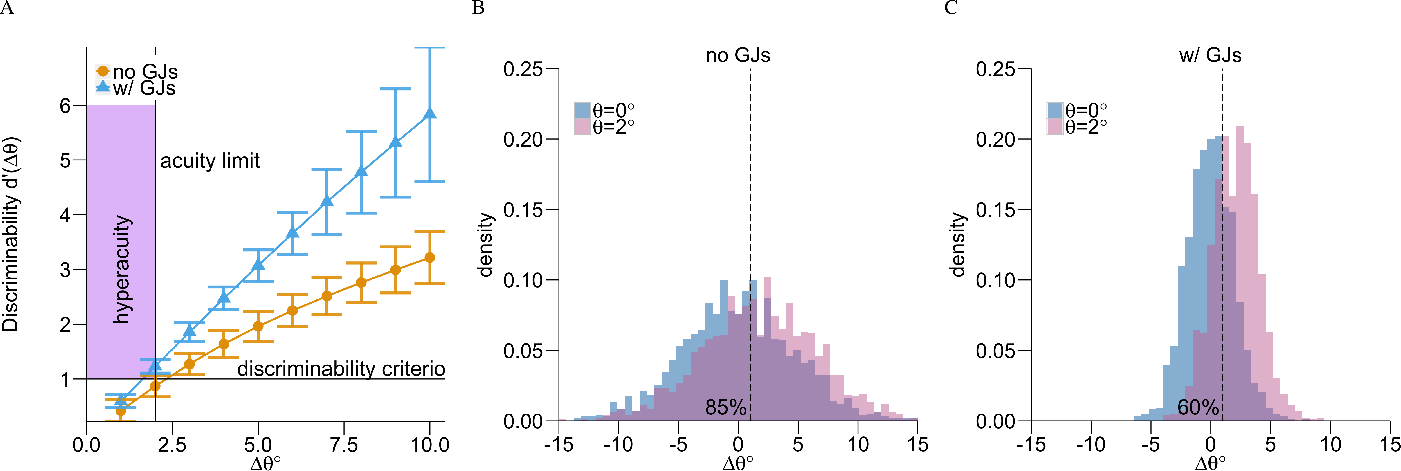

Supplement: S3 Fig — (A) The discriminability, d’, between θ and θ' (θ—θ’ = Δθ) for all axes of rotation as a function of Δθ, with (yellow) and without (blue) GJs. Error bars indicate one standard deviation. Note that only the blue curve intersects the hyperacuity region whereas the orange curve does not. (B) The uncertainty distribution density for θ = 0° (blue histogram) and θ' = 2° (pink histogram) without GJs. The dashed line represents the decision rule, (θ + θ')/2. When a stimulus falls to the left of the dashed line, it belongs to the red distribution; otherwise, to the green distribution. The 85° overlap indicates that this decision rule has a 57% likelihood of being correct (see text). (C) Similar to (B), but with GJs. In this case, the (θ + θ')/2 decision rule has a 70% likelihood of being correct, corresponding to the 60% overlap between the two histograms. (TIF) [file pcbi.1005846.s003.tif]

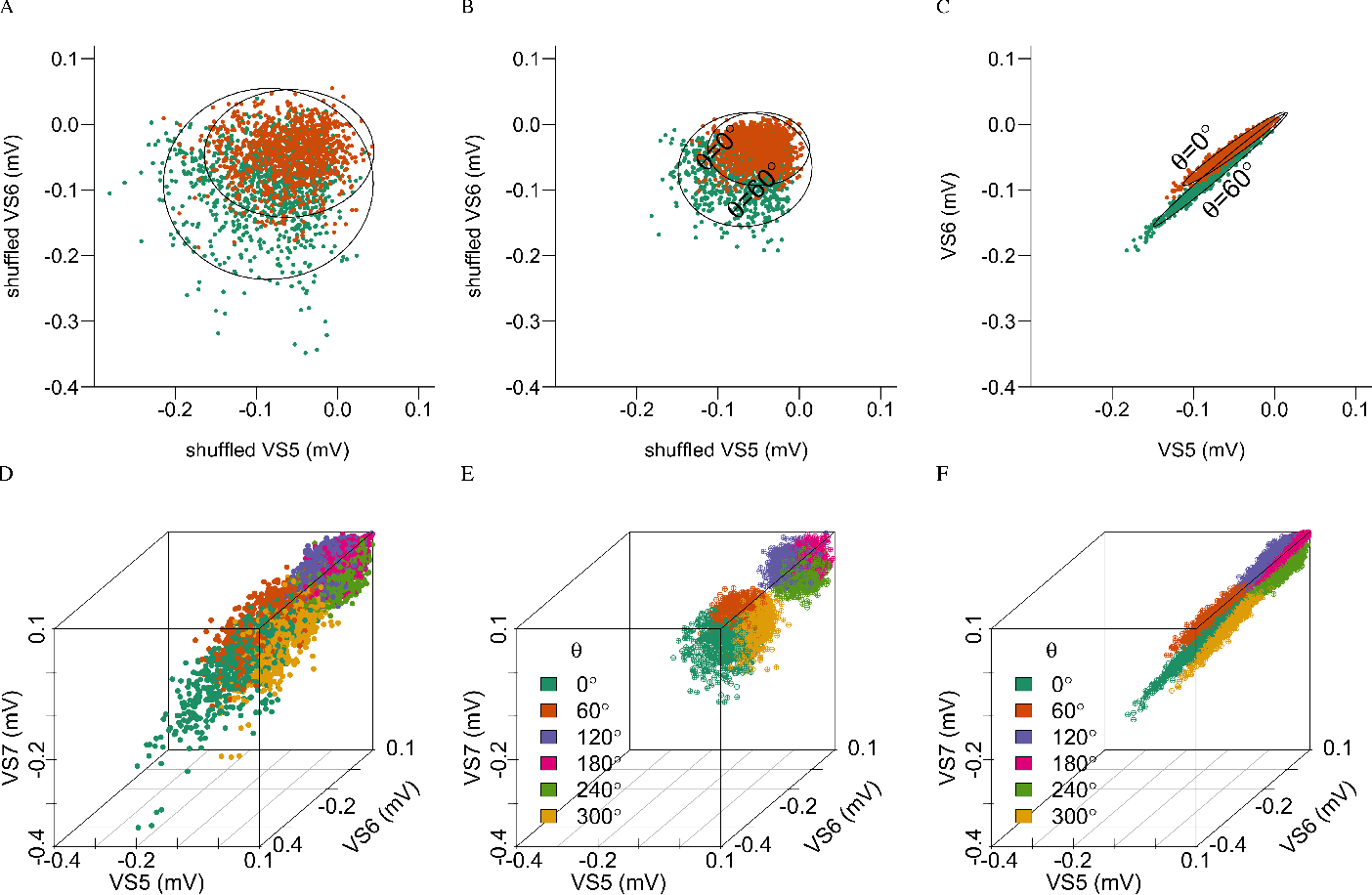

Supplement: S4 Fig — (A) Joint axonal voltage response of VS5 versus VS6 in the absence of GJs. A total of 1000 samples for both θ = 0° (green) and for θ = 60° (red) in response to natural stimuli are shown (see Materials and Methods). Their 95% confidence ellipses are shown in black. (B) Shuffled joint axonal voltages of VS5 and VS6 (95% confidence ellipses shown in black); (C) As in (A) but with GJs = 1 μS. (D) Joint axonal voltages for VS5-6-7 of the left compound eye without GJs for six different axes of rotation (indicated by respective colors). (E) Shuffled joint axonal voltages of VS5-6-7 with the same color code as in (D). (F) Joint axonal voltages for VS5-6-7 with GJs = 1 μS. Shuffled joint axonal voltages of VS 5-6-7 (with GJs) still show capability to cluster different axes of rotations (comparing (E) to (D)) but it is inferior to the case without shuffling (comparing (F) to (E)). (TIF) [file pcbi.1005846.s004.tif]

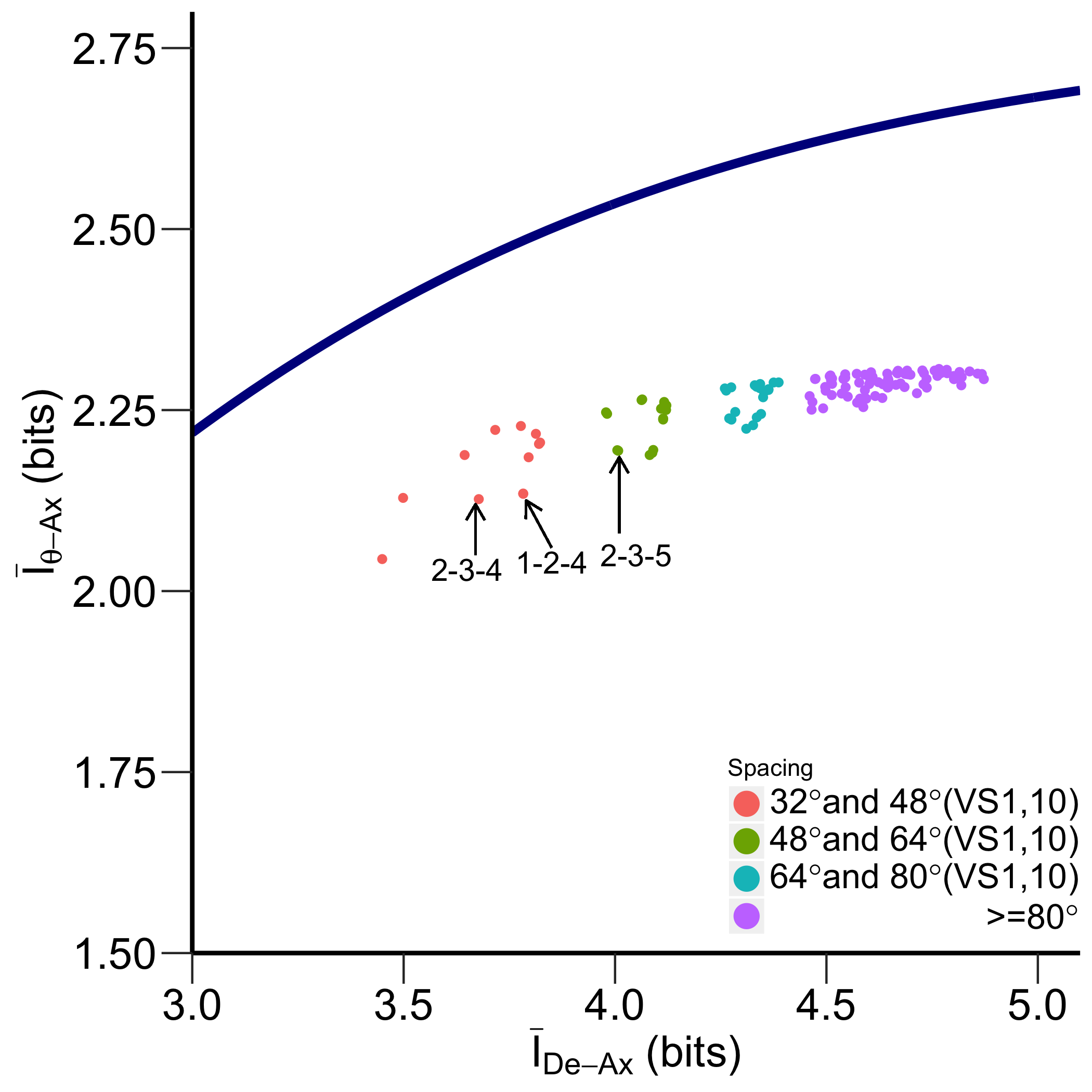

Supplement: S5 Fig — Encoding of triplets for natural stimuli (with GJs), color coded according to the triplet tuning spacing (see text). Note that the e.g., the cluster in red contains both triplets with spacing of 32° as well as triplets with spacing 48° and contain VS1 or VS10. Arrows are pointing to VS 2-3-4, VS1-2-4 and VS 2-3-5, respectively, showing that triplet with boundary VS cells (VS1-2-4 with spacing of 48°) clusters together with VS2-3-4 (with spacing of 32°) rather than with VS 2-3-5 (the cluster in green with spacing of 48°). (TIF) [file pcbi.1005846.s005.tif]

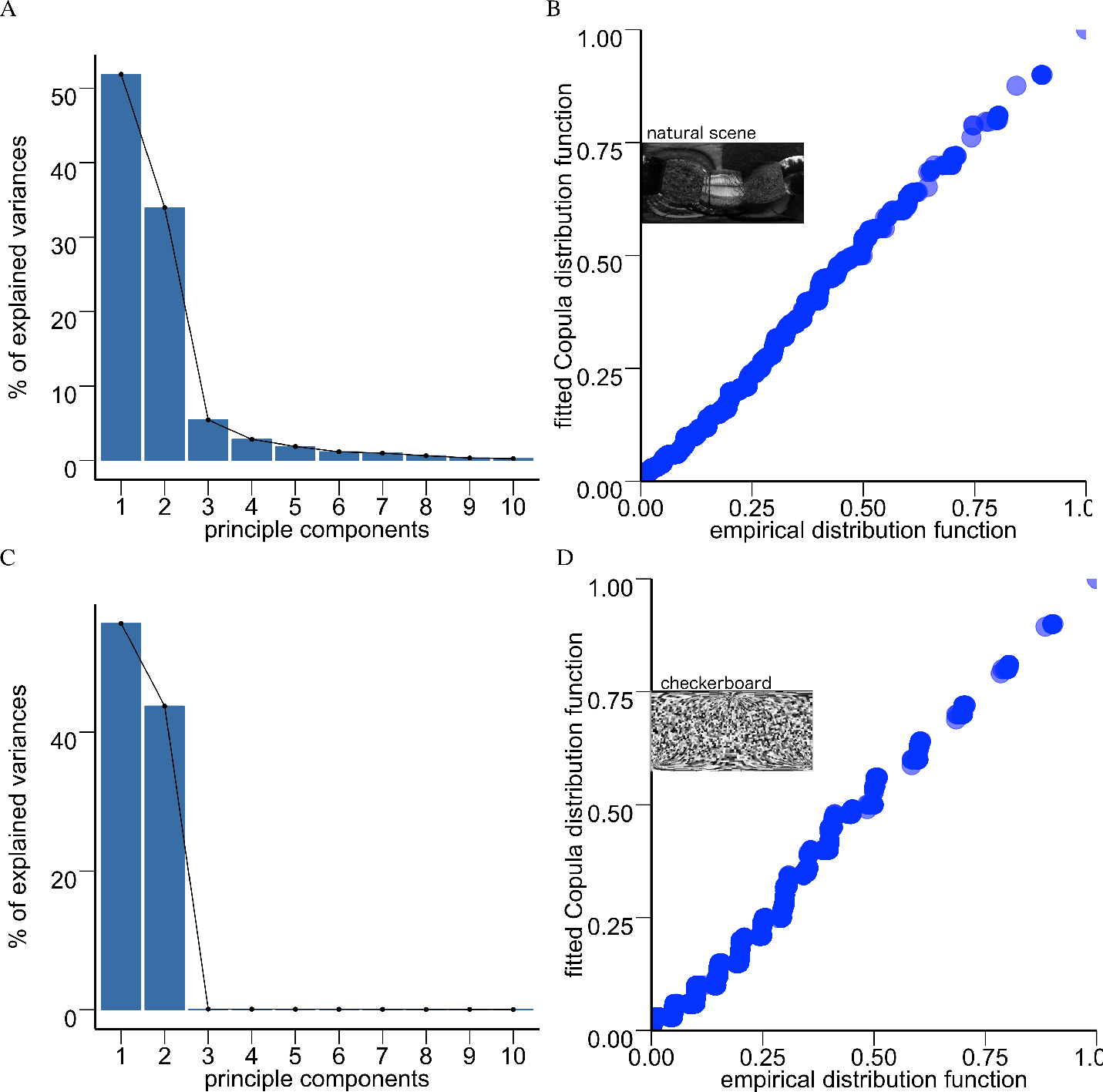

Supplement: S6 Fig — (A) The ten most significant principal components from the currents and the percentages of variance that they explain individually based on the natural stimuli. Note that 90% of the variance can be explained with the two most significant principal components. (B) The quantile-quantile plot the with P(curr,θ), where the current is represented by its two most significant principal components, and θ is represented as (cosθ,sinθ). The points are the quantile values of the empirical copula (x-axis) against the fitted Gaussian copula (y-axis) for 10,000 equally spaced points of the form (0.1 m, 0.1 n, 0.1 p, 0.1 q) with 1 ≤ m, n, p, q ≤ 10. We obtained these values based on 360,000 samples, (1000 samples for each individual axis of rotation between 0° and 360°). (C) Similar to (A) but with the checkerboard stimuli. Note that the first two most significant principal components explained all variance. (D) Similar to (B) but with the checkerboard stimuli. (TIF) [file pcbi.1005846.s006.tif]
