# Supplementary material for: Efficient encoding of motion is mediated by gap junctions in the fly visual system
Source: PLoS Comput Biol. 2017 Dec 4;13(12):e1005846. doi: 10.1371/journal.pcbi.1005846 (PMC5730180; doi:10.1371/journal.pcbi.1005846)
Supplement: S1 Text — (DOCX) [file pcbi.1005846.s007.docx]

**With GJs, encoding by the VS 5-6-7 triplet shows hyperacuity level discrimination**

S3 Fig depicts the discrimination between two stimuli in the checkerboard scene to investigate how GJs affect the acuity of the VS network, with respect to its acuity limit; i.e., the angular distance (2°) between two neighboring LMDs (see **Material and Methods**). This figure shows how the discriminability d' [41]varies as a function of Δθ, in the absence and presence of GJs (in blue and orange, respectively). In the presence of GJs, the representation of the VS 5-6-7 triplet shows discriminability d' > 1 with Δθ =2° (see the blue dot and error bar within the purple shaded area in S3B Fig). Considering that the spacing between two neighboring photoreceptors is 2°, this discriminability indicates that with GJs, the representation of the VS 5-6-7 triplet exhibits hyperacuity.

To understand the implications of hyperacuity, we plotted the uncertainty distributions for θ = 0° and θ' = 2° with (S3B Fig) and without (S3C Fig) GJs. In S3B Fig, there is about 85% overlap between these two distributions, which yields a 57% success rate for discriminating θ and θ' using the discrimination rule: if a sample falls on the dashed line it belongs to θ = 2°; if it falls to the left of the dashed line it belongs to θ =0°. This success rate (57%) is fairly close to the success rate of discrimination by chance; i.e., 50%. As shown in S3C Fig, GJs reduce the overlap between these uncertainty distributions to only 60%, which increases the success rate of discrimination between θ = 0° and θ' = 2° to 70%. This success rate corresponds to a 120% improvement with GJs compared to without GJs.
